# Alginate Materials and Dental Impression Technique: A Current State of the Art and Application to Dental Practice

**DOI:** 10.3390/md17010018

**Published:** 2018-12-29

**Authors:** Gabriele Cervino, Luca Fiorillo, Alan Scott Herford, Luigi Laino, Giuseppe Troiano, Giulia Amoroso, Salvatore Crimi, Marco Matarese, Cesare D’Amico, Enrico Nastro Siniscalchi, Marco Cicciù

**Affiliations:** 1 Department of Biomedical and Dental Sciences and Morphological and Functional Imaging, Messina University, 98100 ME Messina, Italy; gcervino@unime.it (G.C.); lucafiorillo@live.it (L.F.); amoroso.giulia@hotmail.com (G.A.); matamarco94@gmail.com (M.M.); cesaredamico89@gmail.com (C.D.); enastrosiniscalchi@unime.it (E.N.S.); 2 Department of Maxillofacial Surgery, Loma Linda University, Loma Linda, CA 92354, USA; aherford@llu.edu; 3 Multidisciplinary Department of Medical-Surgical and Odontostomatological Specialties, University of Campania “Luigi Vanvitelli”, 80100 Naples, Italy; luigi.laino@unicampania.it; 4 Department of Clinical and Experimental Medicine, University of Foggia, 71122 Foggia, Italy; giuseppe.troiano@unifg.it; 5 Department of Surgical and Biomedical Sciences, Catania University, 95123 Catania, Italy; torecrimi@gmail.com

**Keywords:** impression materials, marine derivates, alginates, marine algae

## Abstract

Hydrocolloids were the first elastic materials to be used in the dental field. Elastic impression materials include reversible (agar-agar), irreversible (alginate) hydrocolloids and synthetic elastomers (polysulfides, polyethers, silicones). They reproduce an imprint faithfully, providing details of a high definition despite the presence of undercuts. With the removal of the impression, being particularly rich in water, the imprints can deform but later adapt to the original shape due to the elastic properties they possess. The advantages of using alginate include the low cost, a better tolerability on the part of the patient, the ease of manipulation, the short time needed for execution, the instrumentation and the very simple execution technique and possibility of detecting a detailed impression (even in the presence of undercuts) in a single step. A comprehensive review of the current literature was conducted according to the PRISMA guidelines by accessing the NCBI PubMed database. Authors conducted a search of articles in written in English published from 2008 to 2018. All the relevant studies were included in the search with respect to the characteristics and evolution of new marine derived materials. Much progress has been made in the search for new marine derived materials. Conventional impression materials are different, and especially with the advent of digital technology, they have been suffering from a decline in research attention over the last few years. However, this type of impression material, alginates (derived from marine algae), have the advantage of being among the most used in the dental medical field.

## 1. Introduction

Irreversible hydrocolloid impressions are a common part of daily practice. Alginate is one of the most frequently used dental materials; the alginate impression is usually performed at the first dental visit and its results are fundamental to forming a first “idea” about the patient’s oral health status. For many years, alginate impression material has been a staple of most dental practice and impression materials are an important consideration for dental clinics even today. Therefore, it is important to understand the material and follow certain fundamental guidelines in order to achieve flawless, predictable impressions and hence avoid repeat impression/restorations. The Food and Drug Administration (FDA) issued a document in 1998, defining a Dental Impression Material as a class II device composed of materials such as alginate or polysulfide intended to be placed on a preformed impression tray and used to reproduce the structure of a patient’s teeth and gums. The device is intended to provide models for studies and for production of restorative prosthetic devices, such as gold inlays and dentures. See 21 Code Federal Regulation CFR Part 872.3660. (FDA Product Code “ELW”). Accordingly, the International Standard for Organization ISO standard, ISO-10993, Part 1 [1,2,3], uses an approach to test selections, which is very similar to the Tripartite Guidance used in the past by the FDA. It also uses a tabular format (matrix) for laying out the test requirements based on the various factors discussed above. The matrix consists of two tables; the Initial Evaluation Tests for Consideration and the Supplementary Evaluation Tests for Consideration. In order to harmonize biological response testing with the requirements of other countries, the FDA has recognized the ISO standard. Reviewers in the Office of Device Evaluation will accept data developed according to ISO-10993, Part 1, with the matrix as modified and presented in Blue Book Memorandum #G95-1 entitled “Use of International Standard ISO-10993, Biological Evaluation of Medical Devices Part-1: Evaluation and Testing.” [1,2,3,4].

Hydrocolloid materials for dental impressions are available in the form of viscous liquids in the “sol” state or in the form of semi-solid substances of a gelatinous consistency. Without a filler, the gel would lack stability and would have a slimy surface covered with synerate exudate. Alginates are salts of alginic acid, a polysaccharide extracted from the cell walls of brown algae (washed, ground and chemically treated, especially the pulp) belonging to the Phaeophyceae family, widespread especially in America [5].

The extracted alginic acid is then converted into a salt (alginate) of sodium, calcium, potassium or magnesium. Although alginate is insoluble in water, its alkaline salts are water-soluble and therefore sodium or potassium alginate is used in the dental field. The production process of sodium alginate from brown algae can be done in two ways; using the calcium alginate method or the alginic acid method. To extract alginic acid, the algae are placed in a sodium carbonate bath, exploiting the solubility of alkaline alginates in water. The alginic acid is recovered from the obtained solution by precipitation with hydrochloric acid or sulfuric acid. The difficulty of the processes lies in the required physical separations, such as in the filtration of muddy residues from viscous solutions or in the separation of gelatinous precipitates that retain a large amount of liquid in their structure, resisting filtration and centrifugation. Alginates are used as thickeners and stabilizers in the food, pharmaceutical and cosmetic industries, are easy to use, low cost, well tolerated by patients, excellent for primary prosthetic, orthodontic and design imprints. They come in the form of a powder to be mixed with water in appropriate doses. Once mixed, the alginate turns into a soft paste that is placed on the tray and introduced into the oral cavity for the detection of the impression. They are irreversible hydrocolloids because the picking reaction is a chemical reaction of irreversible precipitation, therefore they cannot return in sol form using physical means, such as temperature, as with reversible hydrocolloids. The chemical reaction occurs two times: a first phase called ‘slowing’ and a second phase called ‘setting’. Initially the powder is mixed with water and the sodium phosphate reacts with the calcium sulfate to allow an adequate processing time. After the sodium phosphate has reacted, the remaining calcium sulfate reacts with sodium alginate to form an insoluble calcium alginate that forms a gel with water which acts as a catalyst. The alginates available on the market can be of two types: fast setting (hardening time of 1–2 min) or normal setting (setting time between 2–5 min). The setting time depends on the composition (water/powder ratio, where increasing the powder accelerates the hardening reaction) and the temperature at which mixing takes place (the setting time is inversely proportional to the temperature, where the higher the temperature, the lower the setting time and therefore the reaction is faster). Dust tends to lose its organoleptic characteristics when exposed to moisture or heat. To obtain a better product, the alginate must be integrated with the following:

1. Borax, zinc sulphate, and sodium fluoride in order to increase the resistance of the impression and the hardness of the model surface, avoiding the adherence of the impression of alginate to the plaster during the casting of the model.
2. Fossil flour or diatomaceous earth, which has the function of being a filler and also controls the fluidity and the consistency of the mass, making the impression surface smooth and compact.
3. Chemical indicators: These are substances that have the ability to make the material change color as its acidity varies during the gelling reaction [5].

The advantages of the use of alginate are therefore in the low cost, better tolerability on the part of the patient, ease of handling, short execution time, instrumentation and the very simple execution technique, and the possibility of detecting a detailed impression (even in the presence of undercuts) all in a single step. The use of these materials in the dental field is very common due to their low cost. They are generally used as materials for a first study impression with a medical and diagnostic purpose. Some alginates differ from others in terms of quality and therefore can be used for different purposes. Moreover, others have changed setting times so as to reduce the patient’s discomfort [6].

## 2. Materials and Methods

### 2.1. Target Questions

The following spotlight questions were processed following the guidelines, parameters, and possible aims of the Patient Intervention Control Outcome (PICO) study design:

- What is the contribution of marine sciences in the field of medical impression materials?
- Do marine derived materials used for impression techniques make clinical and scientific contributions in dentistry?

### 2.2. Searches

**Scheme 1:**
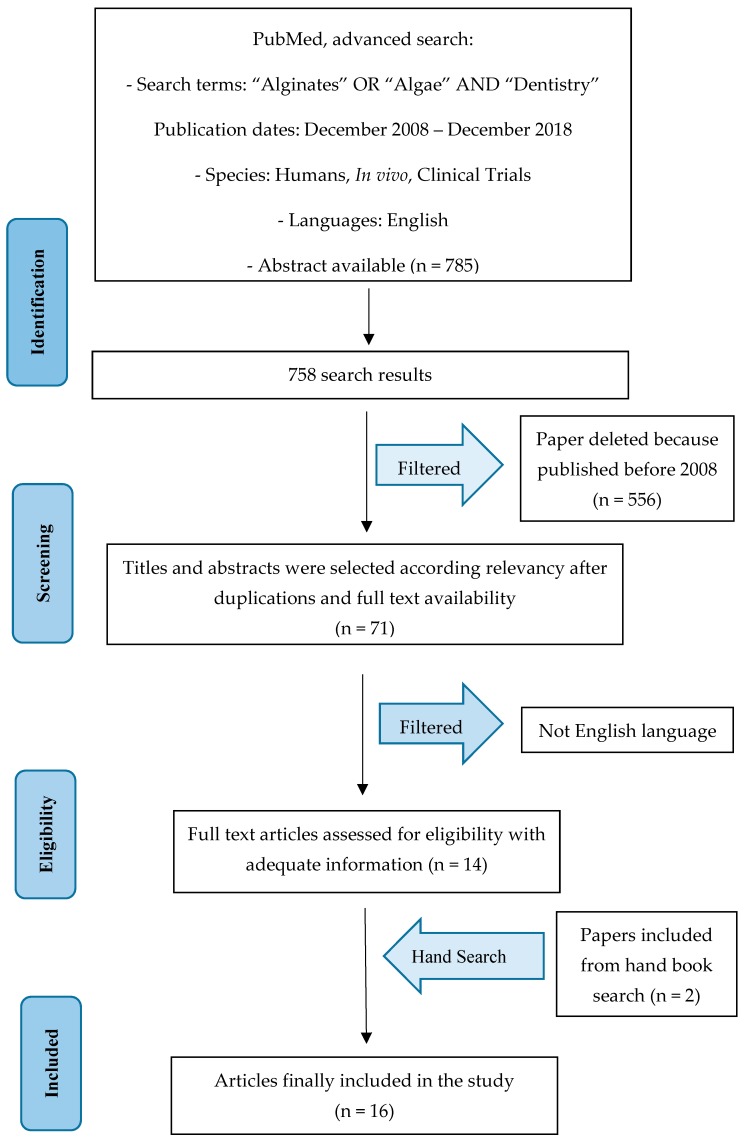
Prisma Flow diagram.

The PubMed–Medline resource database was explored through advanced searches. The keywords and search inquiries used during the primary stage were as follows: “alginates dentistry”, “algae dentistry”. Additionally, manually selected articles were included regarding the eligibility criteria. Scheme 1 represents the flow diagram of the selected studies according to the PRISMA guidelines in accordance with the criteria for the choice of investigated papers.

### 2.3. Data Recorded from the Selected Manuscripts

The Medical Subject Headings (MeSH) were applied to find the keywords used in the present study. The selected key words: “alginates” or “algae” and “dentistry” were recorded for collecting the data.

### 2.4. Selections of the Papers

The manuscripts selected in the present study highlighted clinical researches on humans published in the English language. Letters, editorials, case reports, animal studies and PhD theses were excluded.

### 2.5. Research Classifications

The method of classification included all human prospective and retrospective clinical studies, split mouth cohort studies, case–control papers, and case series manuscripts, published between December 2008 and December 2018, on alginates impression materials used in dentistry.

### 2.6. Statement of the Problem

The sentence case of “impression material” was searched over each selected paper.

### 2.7. Exclusion and Inclusion Criteria

The inclusion criteria applied to the studies were as follows:

- English language.
- Clinical human studies or merchandising updates on alginates impression materials used in dentistry.

The following types of articles were excluded as follows:

- vivo/in vitro studies.
- Studies not relevant to our selected topic.
- Animal studies.
- Medicated impression materials, digital impression techniques, or use of combined alginates.
- Literature review articles published prior to 1 December 2018.
- No access to the title and abstract not in English language.

### 2.8. Strategy for Collecting Data

Following the initial literature search, all article titles were screened to eliminate irrelevant publications, case reports, and animal studies. Next, studies were excluded based on data obtained from screening the abstracts. The final stage of screening involved reading the full texts to confirm each study’s eligibility based on the inclusion and exclusion criteria.

### 2.9. Record of the Extracted and Collected Data Extraction

The results and conclusions of the selected full text papers were used for assembling the data according to the aims and themes as listed.

The following parameters were used for assembling the data, which was then organized according to the following schemes:

“Author (Year)” revealed the first author and the year of publication.

“Result” indicated the field of use for the material, or any strengths and weaknesses therein.

### 2.10. Risk of Bias Assessment

The grade of bias risk was independently considered by the authors and coordinator. The quality of all the included studies was assessed during the data extraction process. The quality appraisal involved evaluating the methodological elements that might influence the outcomes of each study. According to Higgins, this study followed the Cochrane Collaboration’s two-part tool for assessing risk of bias and PRISMA statement [7].

The risk of bias in this “perspective” study was assessed only for the articles from which the discussion was developed. Being a report on alginate impression materials in dentistry and a view on new prosthetic technologies, the risk of bias is minimal on this work.

### 2.11. History of Impression Materials

The development of dental impression materials began in the mid 1800s. Dentists have realized that the construction of a prosthetic restoration required detailed reproducibility of the dental arches of patients, and the construction of plaster models. Beeswax was the first impression material, although the first important signs of evolution of dental impression materials are considered the introduction of trays at the beginning of the 1800s and the invention of gutta–gercha, thermoplastic resins and plaster of Paris. The double technique of impression combined with the concept of functional impression that was established after the mid-1800s, are also identified as fundamental innovations. During the 20th century, advances in material development slowed significantly because most of the current print materials had already been invented. However, the introduction of elastomeric impression materials in the dental prosthesis field that offered the advantages of accuracy and dimensional stability have substantially improved both the accuracy of the impression and the quality of the final restoration. Impression materials are used in many fields, including crafts for model reproduction. Continuous evolutionary drive lead to the discovery and use of hydrocolloids. Historically, alginate has been considered the first impression material to be valid from a clinical point of view and with regard to invasiveness of the patient [8].

Reversible hydrocolloids have been used since 1937 and irreversible hydrocolloids since 1947 for the creation of dental impressions. Alginates are of plant origin and are extracted from marine algae. Impression techniques of the 20th century were similar to those of today. The dimensional accuracy of irreversible hydrocolloid reproduction has been demonstrated since the 1950s. In most clinical cases, the new alginates used in metal trays are as accurate as the “old” impression materials. Combination materials are less accurate when many prosthetic pillars need to be reproduced. The use of perforated or non-perforated metal trays with alginates has no effect on accuracy. Although alginates used in disposable plastic trays can cause serious inaccuracies. In tight spaces with undercuts, errors can still be detected during the impression taking [9,10]. Irreversible alginates are made of Ester salts of alginic acid for 15% of products, calcium sulphate that works as a reactor for 16%, zinc oxide for 4%, potassium titanium fluoride for 3%, diatomaceous earth for 60% and sodium phosphate and coloring or flavoring agents for 2%. The setting reaction is a chemical reaction between Sodium Alginate and Calcium Sulphate, where:

(1) $$2{\mathrm{Na}}_{3}{\mathrm{PO}}_{4}+3{\mathrm{CaSO}}_{4}\to {\mathrm{Ca}}_{3}{\left({\mathrm{PO}}_{4}\right)}_{2}+3{\mathrm{Na}}_{2}{\mathrm{SO}}_{4}$$

This reaction (1) can be retarded with Calcium Phosphate, which acts as a retarder, thereby increasing the setting time and obtain a type I or type II setting time:

- Type I (fast set): 1–2 min.
- Type II (normal set): 2–4.5 min [11,12].

### 2.12. Use of Alginates in Dentistry

Dental impression techniques are variable, depending on which material is used. The irreversible hydrocolloids, which are the most commonly used, are a mixture of manual or mechanized techniques through the union of a powder and water. Alginate impression materials are easy to use, less expensive and have more rapid setting times. The reaction time and therefore the setting time can be controlled with the temperature of the water used. They are slightly flavored and in other cases their color turns according to the phases of the chemical reaction. Their disadvantages include less accurate reproduction of elastomeric impression materials, and poor dimensional stability for complicated jobs. Moreover, with these materials it is possible to produce only one plaster model. It is important to select the correct tray for the dental arch, which should be perforated. Alginic adhesives can be used in addition to perforations for the retention of alginate in the tray. The use of alginic adhesives exceeds alginate adhesives, which are available as paints or spray-ons. After applying an alginate adhesive, it is allowed to dry for 5 min. The changes to the tray can be made with wax or with silicone.

Usually a powder measuring cup is supplied with the powder impression material, and a cylindrical plastic measuring cylinder for water. Some water supplies contain large amounts of minerals that can affect the accuracy and setting of the alginate. In these cases, it is possible to use distilled or demineralized water. The mixing is started by adding a quantity of water into the bowl and then follows the addition of a proportioned quantity of powder. The colder water can be used if a longer working time is desired. The variation of the setting time also depends on the amount of water. Mixing must be quick with a wide-blade spatula. The resulting mixture should be creamy in consistency but should not drip off the spatula when it is lifted from the bowl [13,14].

For non-exemplary bowls, it is necessary to perform an accurate drying in order to avoid bubbles. Alginate radicals in the impression material form chemical bonds with enamel hydroxyapatite crystals and therefore, defects in the impression. Once the material is placed in the tray, this is inserted into the patient’s arch. Light pressure is applied, and held in place. Soft tissues, especially labial flanges, should be relieved and manipulated so that alginate can flow into the grooves and record details. Once set, the fingerprint must be removed with a quick snap. The impression should not be rocked or twisted before or during removal of the impression.

The alginate impression should be washed with a jet of water, disinfected and dried until the gloss disappears. Store with gauze and leave in a plastic bag with a waterproof zip closure until the model is made [15,16].

Surely one of the most debated aspects in the field of impression materials, in addition to resolution, is the stability of the impressions over time. This is one aspect to be improved upon [17]. These impressions, once taken, cannot be stored for a long time and will undergo a volumetric change based on many environmental factors, which lead to inaccuracies in dental impressions. The last alginates allow one to personalize chair times, unlike during the first formulations, improving stability over time, higher resolution, and ease of use [18,19].

The hydrocolloids that have been widely discussed here are not the only impression materials but they are the only ones of marine derivation. These materials are evolving over time, greatly improving in their quality and fidelity. The other materials are:

Thermo-plastic pastes, which are made of gutta-percha, gum, talc, wax, dyes and other materials. They soften easily and once they have reached the plastic state (from 45 to 75 °C, according to the types) they are placed in the impression holder. They are used exclusively for single prints with a little copper ring, or in some cases for the border. Among the irreversible materials we have elastomers, which are rubbery materials with excellent elasticity so they perfectly reproduce all the undercuts of the oral cavity (for this reason they are widely used). They are composed of two materials: the basic paste and the fluid paste. They come in tubes and are mixed with the appropriate catalysts. The “double impression” technique is then used. Elastomers are more stable than alginates but they must also be developed quickly [20]. Other impression materials that have been on the market in recent years are “digital” materials. Fingerprinting techniques promise to replace all other materials in a few years due to their rapid evolution and push from the industries [21].

Alginates and marine derivatives of algae are also used for other purposes in the medical field. Although this is not the topic of the study, it is at least obligatory to mention them briefly. Alginates can be used primarily to take impressions even in contact with other structures such as bone structures, both in the maxillofacial region and in other regions in orthopedics [22]. This material must be completely removed because it can go against an inflammatory reaction from the tissues [23]. It is useful to have stone models in the surgical field, this has over time been replaced by stereolithography but a stone model of bone thicknesses and mucosa made with alginate impression materials is very useful [24], even in the case of bone deformities or associated neoformations with dental elements [25]. The alginic materials also have an antacid action and for this reason, antacid drugs have been developed for gastric syndromes [26]. The chemical structure of alginates is also similar to some drug formulations including that of heparin, and may have a similar effect [27]. Knowledge of alginate chemical structures and functionality are known to be important parameters in the design of alginate-based matrices for cell cultures [28]. Alginate oligosaccharides with different bioactivities can be prepared through the specific degradation of alginate-by-alginate lyases. Therefore, alginate lyases that can be used to degrade alginate under mild conditions have recently attracted public attention. Although various types of alginate lyases have been discovered and characterized, few can be used in industrial production [29]. Alginic acids and alginates in some cases, being approved by the FDA, and biocompatible, are loaded with drugs so as to work as carriers. in one study, these drugs were used for juvenile idiopathic diseases, including juvenile idiopathic arthritis [30,31].

## 3. Results

### Manuscript Collection

The manuscript choice and process for analyzing the data followed the PRISMA flow diagram (Scheme 1). The first electronic and manual search was performed on Pubmed, Medline and Oral Sciences Source, which resulted in a total of 785 papers. 556 papers were excluded because they were published prior to 1 December 2008. Then, another 485 papers were excluded in the revision because they were not available in full text versions. At this point, titles and abstracts were evlauated and only the English texts were kept. Fourteen articles were selected as having significant data regarding the “alginate impression materials used in dentistry” topic. Two articles were added from books or external journals (Table 1).

## 4. Discussion

In the last few decades, the use of this kind of materials has found different applications related to several medical fields. The food industry has widely applied alginates as additives because of their gelling, viscosity, and stabilizing properties. Therefore, the ingestion of sodium alginate (SA) and subsequent gelation in the stomach seems to reduce human appetite in acute settings. An important rheological property of fibers within the intestine is viscosity, which is thought to account for beneficial physiological responses in relation to appetite regulation, as well as glycemic and lipidemic control [32,33,34]. Other investigations within the pharmacological field have demonstrated how alginate-antacid formulations can decrease post-prandial symptoms by neutralizing the acidity of gastric contents by forming a gel-like barrier to displace the “acid pocket” from the esophagogastric junction and protect the esophageal and gastric mucosa [34,35]. Alginates have also been used for thickening gel in forming, and stabilizing agents, as alginate can play a significant role in controlled-release drug products. Specifically, Alginate has also been widely exploited in many drug delivery applications in combination with chitosan because the combination forms ionic complexes. Another field of application related to cell cultures, is that alginate gels can be used as a model system for mammalian cell culture in biomedical studies [36]. Despite recent progress, treatment of bone injuries is still often limited due to poor healing. Alginate gels have found some potential in bone regeneration in the delivery of osteo-inductive factors, bone-forming cells, or a combination of both. Recently, alginate gels have also been actively investigated for their ability to mediate the regeneration and tissue engineering of different tissues and organs, including skeletal muscle, nerves, the pancreas, and liver. Current strategies for skeletal muscle regeneration include cell transplantation, growth factor delivery, or a combination of both approaches. Alginate gels have some potential in these strategies [37,38].

The studies selected in this work are different, and all of them concern alginate impression materials. These materials as already explained in the previous chapters, have their merits and defects. Now they will be analyzed in more detail. One of the most debated aspects is that of the material’s stability over time once the impression is taken. Cesur et al. says that plaster models obtained with alginates after different storage times, demonstrate statistically significant differences between the models, but measurements of the dental arch perimeter do not differ [18]. The dimensional stability of different irreversible hydrocolloids over time was evaluated by Garrofè et al. in 2015. There are differences over time in the alginates considered (LASCOD and ZHERMACK), measured by a photograph at different time intervals [39,40]. Another study compared the surface detail reproduction and the dimensional accuracy of stone models obtained with different alginate impression materials. In this case there was no statistically significant difference in the mean dimensional accuracy values after five days [41]. The fourth study that evaluated deformation, evaluated 90 alginate impressions using different alginates. The impressions were stored in a sealed plastic bag without a damp paper towel for up to 120 h and poured with type III dental stone. Immediate pouring of the alginate impressions provides the highest accuracy regarding teeth and tissues [42].

**Figure 1:**
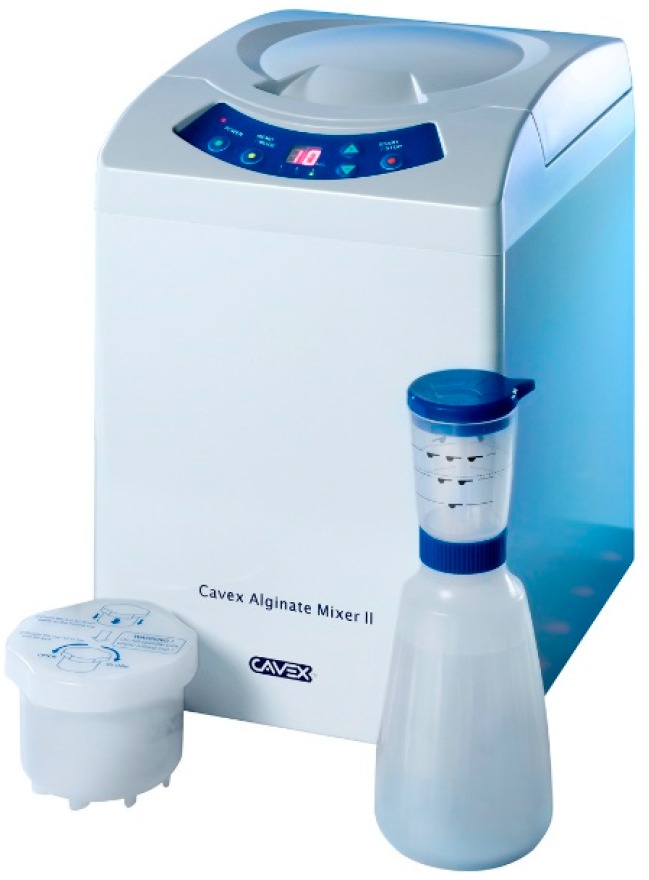
Alginate automixer Cavex^®^, for alginate mixing.

**Figure 2:**
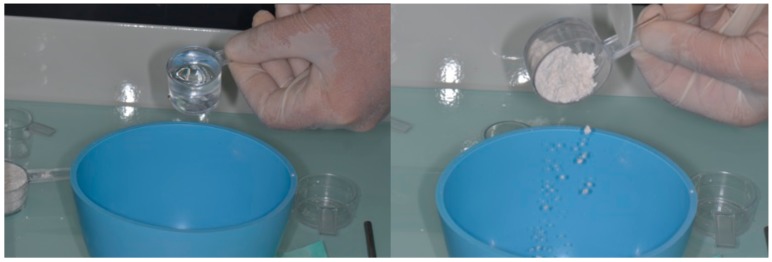
Manual alginate impression material mixing, water and powder. Courtesy Prof M.C.

**Figure 3:**
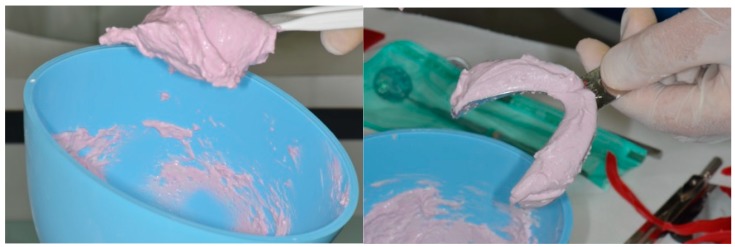
Manual alginate impression material mixing. Alginate on dental impression tray. Courtesy Prof M.C.

Other studies take into consideration the tray design and the impression material technique, or pressure during setting. In this case Inoue et al. consider three types of impression materials, polyvinylsiloxane elastomer, polyether elastomer, and alginate (Figure 1, Figure 2 and Figure 3). The pressure during setting had no significant effect on precision, but an excessive pressure can be alleviated with escape holes and relief on the tray [43]. Hyde et al. in 2014 recruited 85 patients and performed an Randomized Controlled Trial (RCT) using alginate or silicone impressions for complete denture creation. Patients preferred dentures made from silicone impressions (67.9%) compared to alginate impressions. Both dentures were satisfactory but there is significant evidence that dentures made from silicone were preferred by patients [44]. Surface detail and dimensional stability were tested by one caliber and a profilometer. The first group had a contact time between impression material and modeling material of 1 h and group 2 had a 12 h contact time. The study showed a significant difference between groups, where the 12 h time of contact was not recommended because it influences the quality of the plaster cast [45].

The dimensional stability of alginate was both significantly time and temperature dependent. A humid environment and 4 °C temperature can delay pouring [46]. Twenty-five impression materials were observed in the Fonseca et al. study, evaluating radiodensity and comparing them to human and bovine enamel and dentine with the aim of detecting small fragments left inside gingival sulcus or root canals. The persistence of these materials in the gingival sulcus, especially in the case of implants or implants where self-cleaning can be affected, can lead to periodontal inflammation, peri-implantitis and other problems. In the case of implant-prosthetic rehabilitations it is essential to use these materials for rehabilitation [47]. The interface between the impression material and the tooth structure, dentin or enamel can be evaluated radiographically, just as it is done between cements and dental structures [48]. Polysulfides showed high values of radiodensity, comparable to human enamel, but not bovine. Human dentin was similar to heavy-body silicon [49].

Other studies investigated the disinfection of dental impression materials. One of them was the Iwasaki et al. study that evaluated the surface roughness observed on the Scanned Electron Microscopy (SEM) after a sodium hypochlorite solution was applied for up to 10 min with no adverse effect, in contrast to 1 min of ortho-phthalaldeyde, which caused deterioration [50]. Hiraguchi et al. evaluated stone model distortions using a hypochlorite solution of less than 15 µm [51], where no deformations or dimensional changes were present using spray disinfectant with 1% hypochlorite solution and 2% glutaraldehyde solution [52]. According to the manufacturer, the disinfectant solutions used for dental impressions diffuse into alginate mass deeper than oral cavity fluids at the time of impression taking [53].

One of the aspects considered at the end of the discussion is the cost of materials. According to Hulme et al., silicone group as compared to alginate costs are higher but had a greater mean Oral Health Impact Profile - Edentulism (OHIP-EDENT) gain. The additional cost of using silicone was 3.41 £ per OHIP-EDENT point. Silicone impressions for complete dentures, improves the patients’ quality of life for an extra cost of 30 £ [54]. The alginates are therefore less expensive and much more accessible, especially where developing communities in need of prosthetic rehabilitations are concerned.

### Limitations

This work is a collection of studies on the use of alginates in the dental field. The aim is to open up new perspectives on the evolution of this material and to give an account of the situation surrounding the technology of marine derived materials, such as impression materials, bone regeneration materials or periodontal therapy [55]. The risk of bias of this study as already mentioned is minimal, as the outlook is towards future prospects for the materials, and is based on the characteristics already known.

## 5. Conclusions

Technological innovations are taking hold in the world of dentistry. Intraoral scanners and 3D printers, in fact, are increasingly used by dentists. Digital technology represents a revolution both in diagnosis and in therapeutic planning. Innovations of the digital era allow for an ever greater speed of intervention, high precision and personalization of care, with a significant reduction in costs. Such factors that will revolutionize the way of working in dental specialties. In the field of diagnostic images, relevant and specific accurate comparisons have been made. Specifically, in the dental filed it is possible to better evaluate the anatomical position of the impacted teeth, highlighting noble anatomical structures like maxillary sinus or the alveolar dental nerve in a three dimensional position. This theme is fundamental for the design of prosthodontic rehabilitations, given the opportunity to see the bone volume before planning the surgery. As with orthodontic rehabilitation, 3D printers, which surely represent the future, will allow us to make individual orthodontic devices, such as attacks with increasingly better materials. One important technological innovation is less invasive techniques. An example is the intraoral scanner that looks like a toothbrush. This tool will transform the visit to the dentist into a game for young patients who will be able to monitor the images of their teeth directly on the computer screen. New technologies will also produce increasingly less visible dental devices, which by using innovative materials, will offer clear improvements to the quality of life and social relationships which are so important especially for adolescents. And even before starting, thanks to 3D digital imaging technology, the new devices will allow you to see the virtual results and the treatment plan so that you know in advance what the teeth will look like at the end of the treatment. Despite the fact that the future of dentistry is represented by digital methods, the use of alginates, given the cost and simplicity of use always forms the majority. The cost of digital techniques is still very high, and not all dental technicians are able to interface with them. It’s a change that will surely happen, but it will take time. Alginates are excellent materials as a first impression as they are also minimally invasive for patients. They manage to get us a plaster model for a first evaluation of the rehabilitation of our patient in the prosthetic, surgical or orthodontic fields.

As for the future outlook, alginate materials and derived, with regard to gel formation ability, mechanical strength, and interactions with cell via bio-adhesive bonds, and are considered to be a promising material for cell and tissue culture and have been employed in 3D systems [56]. 3D material based on ionically gelled and dried Alginate (ALG) macroporous scaffolds creates favorable conditions for cellular attachment, proliferation, and differentiation. ALG scaffolds are able to turn into hydrogels upon rehydration following cell seeding.

Certainly improving the quality and definition of these materials would be possible to expand their use with benefits for patients, given their reduced setting time and their single-footprint technique, and still provide benefits for dentists. The prospect is that these materials will continue to evolve as has happened since the 40s, thus producing high-performing impression materials. Marine resources are easily accessible and the study of these could also lead to the discovery of new marine derived materials.

## Figures and Tables

**Table 1:** Studies taken into consideration and field of interest.

Reference	Authors	Title	Results	Years
[5], pp. 154–196	Spoto G.	Title of chapter. In *Materiali e Tecnologie Odontostomatologiche*	Alginates features	2013
[5], pp. 150–153	Spoto G.	Title of chapter. In *Materiali e Tecnologie Odontostomatologiche*	Alginates impression techniques	2013
[18]	Cesur et al.	Evaluation of digital model accuracy and time-dependent deformation of alginate impressions.	Deformation and alginates storage	2017
[39]	Garrofé et al.	Linear Dimensional Stability of Irreversible Hydrocolloid Materials Over Time.	Deformation and alginates storage	2015
[40]	Guiraldo et al.	Influence of alginate impression materials and storage time on surface detail reproduction and dimensional accuracy of stone models.	Deformation alginates storage,	2015
[42]	Rohanian et al.	Effect of storage time of extended-pour and conventional alginate impressions on dimensional accuracy of casts.	Deformation and alginates storage and alginates impression detail	2014
[43]	Inoue et al.	Influences of differences intray design and impression material on impression pressure at edentulous mandible.	Alginates impression techniques	2017
[44]	Hyde et al.	A randomised controlled trial of complete denture impression materials.	Alginates impression techniques	2014
[45]	Marquezan et al.	Does the contact time of alginate with plaster cast influence its properties?	Alginates impression techniques and dental impression material costs	2012
[46]	Farzin et al.	Effect of pouring time and storage temperature on dimensional stability of casts made from irreversible hydrocolloid.	Alginates impression detail and deformation	2010
[49]	Fonseca et al.	Radiodensity evaluation of dental impression materials in comparison to tooth structures.	Alginates impression radiodensity	2010
[50]	Iwasaki et al.	Effects of immersion disinfection of agar-alginate combined impressions on the surface properties of stone casts.	Alginates deformation and deterioration during disinfection	2016
[51]	Hiraguchi et al.	Effect of immersion disinfection of alginate impressions in sodium hypochlorite solution on the dimensional changes of stone models.	Alginates deformation and deterioration during disinfection	2012
[52]	Hiraguchi et al.	The influence of storing alginate impressions sprayed with disinfectant on dimensional accuracy and deformation of maxillary edentulous stone models.	Alginates deformation and deterioration during disinfection	2010
[53]	Surna et al.	*In vitro* investigation of the integration depth of oral fluids and disinfectants into alginate impressions.	Alginates deformation and deterioration during disinfection	2009
[54]	Hulme et al.	Cost-effectiveness of silicone and alginate impressions for complete dentures.	Impression materials costs	2014
